# Overexpression of *OAS* Genes in Severe COVID-19: A Cross-Sectional Study of Hospitalized Patients Infected with Delta and Omicron Variants

**DOI:** 10.3390/jcm15062189

**Published:** 2026-03-13

**Authors:** Cristian Oswaldo Hernández-Ramírez, Hazael Ramiro Ceja-Gálvez, Kevin J. Arellano-Arteaga, Elvira Miramontes-Luna, Jorge Hernández-Bello, Pablo Michael Navarro-Rodríguez, Francisco Javier Turrubiates-Hernández, Oliver Viera-Segura, Ferdinando Nicoletti, José Francisco Muñoz-Valle

**Affiliations:** 1 Instituto de Investigación en Ciencias Biomédicas (IICB), Centro Universitario de Ciencias de la Salud, Universidad de Guadalajara, Guadalajara 44340, Mexico; oswaldo.hernandez@academicos.udg.mx (C.O.H.-R.); hazael.ceja@academicos.udg.mx (H.R.C.-G.); jorge89_5@hotmail.com (J.H.-B.); pablo.navarro9219@alumnos.udg.mx (P.M.N.-R.); francisco.turrubiates@academicos.udg.mx (F.J.T.-H.); oliver.viera@academicos.udg.mx (O.V.-S.); 2 Doctorado en Ciencias Biomédicas, Departamento de Fisiología, Centro Universitario de Ciencias de la Salud, Universidad de Guadalajara, Sierra Mojada No. 950, Col. Independencia, Guadalajara 44340, Mexico; 3 Servicio de Medicina Interna, Hospital Civil de Guadalajara “Dr. Juan I. Menchaca”, Guadalajara 44340, Mexico; kevin.arellano@academicos.udg.mx (K.J.A.-A.); miramonlesle@hotmail.com (E.M.-L.); 4 Department of Biomedical and Biotechnological Sciences, University of Catania, 95123 Catania, Italy; ferdinic@unict.it

**Keywords:** *OAS* genes, interferon response, severe COVID-19, Delta variant, Omicron variant, clinical outcome

## Abstract

**Background/Objectives**: COVID-19 is an infectious disease caused by SARS-CoV-2. The innate immune response constitutes the first line of antiviral defense. Notably, interferon-stimulated genes, such as those belonging to the oligoadenylate synthetase (*OAS*) family, have been implicated in host susceptibility and the response to SARS-CoV-2 infection. However, the extent to which *OAS* gene expression varies across SARS-CoV-2 variants remains insufficiently characterized. Its relationship with clinical outcomes in hospitalized patients is also unclear. This study aimed to evaluate *OAS* gene expression and its association with inflammatory markers and clinical outcomes in patients with severe COVID-19. **Methods**: An analytical cross-sectional study was conducted in patients hospitalized with severe COVID-19 between October 2021 and February 2022. SARS-CoV-2 infection and viral variants were prescreened at admission. Clinical parameters were recorded, including serum cytokine levels (IL-1β, IL-6, IL-8, MCP-1, IFN-α, IFN-β, IFN-γ, and TNF-α), and hematological indices (neutrophil-to-lymphocyte ratio and platelet-to-lymphocyte ratio). The COVID-GRAM risk score, treatment, and hospitalization outcomes were recorded. Relative mRNA expression of *OAS1*, *OAS2*, *OAS3*, and *OAS-L* was quantified by quantitative PCR, using *TaqMan* probes. **Results**: A total of 76 hospitalized patients with severe COVID-19 were included. In-hospital mortality was 32.9%, with a predominance of male patients (60%). Nearly 50% of non-survivors were infected with the Omicron variant. *OAS1*, *OAS2*, and *OAS3* were overexpressed in hospitalized patients with severe COVID-19 compared with healthy subjects (HS) (log_2_ fold change [95% CI]: *OAS2*: 4.312 [4.161–4.602], *OAS3*: 1.660 [1.485–1.916]; *p* = 0.0040 for both). Expression of *OAS2* and *OAS3* was significantly increased in survivors compared with HS (log_2_ fold change: 4.312 [4.161–4.602] and 1.711 [1.485–1.990], respectively; *p* = 0.0009 and *p* = 0.0025). Similar increases were observed in non-survivors (4.554 [4.251–4.743] and 1.640 [1.081–2.301], respectively; *p* = 0.0002 and *p* = 0.0061) compared with HS. **Conclusions**: *OAS* genes, particularly *OAS2* and *OAS3*, are overexpressed in severe COVID-19. This upregulation was comparable between Delta and Omicron infections, suggesting that the activation of this antiviral pathway is driven more by disease severity than by the specific viral variant.

## 1. Introduction

Coronavirus disease 2019 (COVID-19), caused by severe acute respiratory syndrome coronavirus 2 (SARS-CoV-2), has represented an unprecedented global health challenge since its emergence. The public health emergency was officially declared over on 5 May 2023, after approximately 765.2 million confirmed cases worldwide. However, the virus continues to circulate globally. Indeed, during the last month of 2025, more than 57,000 positive cases were reported worldwide, underscoring the persistent epidemiological and clinical relevance of SARS-CoV-2 infection [1,2]. Despite improvements in vaccination coverage and clinical management, incidence rates have declined. However, severe COVID-19 remains clinically heterogeneous, with multiple demographic, immunological, and virological determinants influencing disease progression and fatal outcomes among hospitalized patients [3,4,5,6].

The clinical variability of COVID-19 has been strongly influenced by viral variants, particularly variants of concern (VOCs) designated by the World Health Organization (WHO), such as Delta (B.617.2) and Omicron (B.1.1.529), highlighting the complexity of COVID-19 pathogenesis. All VOCs have demonstrated enhanced replication capacity compared with the ancestral virus, suggesting increased replication efficacy [7,8,9]. The Delta variant has been associated with increased transmissibility and disease severity, whereas Omicron, despite its enhanced immune evasion, has generally been associated with milder clinical outcomes [10,11,12,13].

During periods of variant transition, however, epidemiologic surveillance data indicate that multiple SARS-CoV-2 lineages may co-circulate at the community level. This co-circulation may introduce heterogeneity when comparing variant-specific clinical or immunologic outcomes. In addition, sequence changes in emerging sublineages may affect RT-PCR assay performance and reduce the predicted sensitivity of certain diagnostic assays [14,15].

Innate immune activation plays a pivotal role in shaping the early antiviral response to SARS-CoV-2 [16,17,18,19]. All VOCs have demonstrated the ability to activate—and in some cases partially evade—double-stranded RNA (dsRNA)-induced cellular responses, including interferon (IFN) signaling pathways [20,21]. IFN-mediated antiviral defense limits virus replication through the induction of a broad range of interferon-stimulated genes (ISGs). Among these, the oligoadenylate synthetase (*OAS*) family represents a key antiviral mechanism, acting as cytosolic dsRNA sensors that detect viral replication in host cells and promote viral RNA degradation through activation of latent RNase L, thereby limiting virus replication [22,23].

The 2′,5′-oligoadenylate synthetase enzymes are nucleotidyltransferases encoded by *OAS* genes, originally identified as part of the ISGs repertoire [24]. The human genome contains four *OAS* family members—*OAS1*, *OAS2*, *OAS3*, and *OAS-L*—located on chromosome 12q24.13. Growing evidence suggests that differential expression and genetic regulation of *OAS* genes may influence susceptibility to infection through IFN-induced antiviral mechanisms [25,26,27,28].

Additionally, markedly low levels of IFN-α and IFN-β have been reported in SARS-CoV-2 infection, accompanied by an exacerbated inflammatory response, which facilitates sustained viral replication and prolonged immune activation, thereby driving disease severity [29]. Similarly, transcriptomic analyses have demonstrated that patients with severe COVID-19 exhibit a delayed and dysregulated IFN response, characterized by partial induction of ISGs despite reduced circulating levels of type I IFNs [30].

Although most infected individuals develop mild to moderate disease, a significant proportion progresses to severe or critical illness, characterized by systemic inflammation and high mortality rates [16]. Consistent with disease severity, COVID-19 is associated with immune dysregulation. This includes excessive production of proinflammatory cytokines and chemokines. Elevated circulating levels of IL-6, IL-1β, IL-8, TNF-α, and MCP-1 have been consistently associated with acute respiratory distress syndrome (ARDS), multiorgan dysfunction, and sepsis [31,32].

Recent studies have reported genetic regulation of *OAS* genes in SARS-CoV-2 infection [33,34]; however, the clinical impact of *OAS* gene expression remains incompletely understood. Moreover, studies directly comparing *OAS* gene expression profiles across different SARS-CoV-2 VOCs and their association with mortality—particularly in hospitalized patients from Latin-American populations—are limited [35,36]. Therefore, the aim of this study was to investigate the association between the expression of *OAS1*, *OAS2*, *OAS3*, and *OAS-L*, cytokine levels, and clinical outcomes in patients with severe COVID-19 infected with the Delta and Omicron variants.

## 2. Materials and Methods

### 2.1. Study Design

A cross-sectional study was conducted at a tertiary-level referral hospital in Jalisco, Mexico, within the Internal Medicine Department of the Hospital Civil de Guadalajara “Dr. Juan I. Menchaca”. Patients were consecutively recruited between October 2021 and February 2022, corresponding to periods during which the Delta and Omicron variants were predominant in Mexico [37].

### 2.2. Study Participants

Mexican adult hospitalized patients (aged > 18 years) of both sexes with a positive quantitative PCR (qPCR) test for SARS-CoV-2 were included. Patients were classified as having severe COVID-19 according to World Health Organization (WHO) criteria. This included oxygen saturation (SaO_2_) < 90%, respiratory rate > 30 breaths/min, and signs of severe respiratory distress [38].

**Figure 1:**
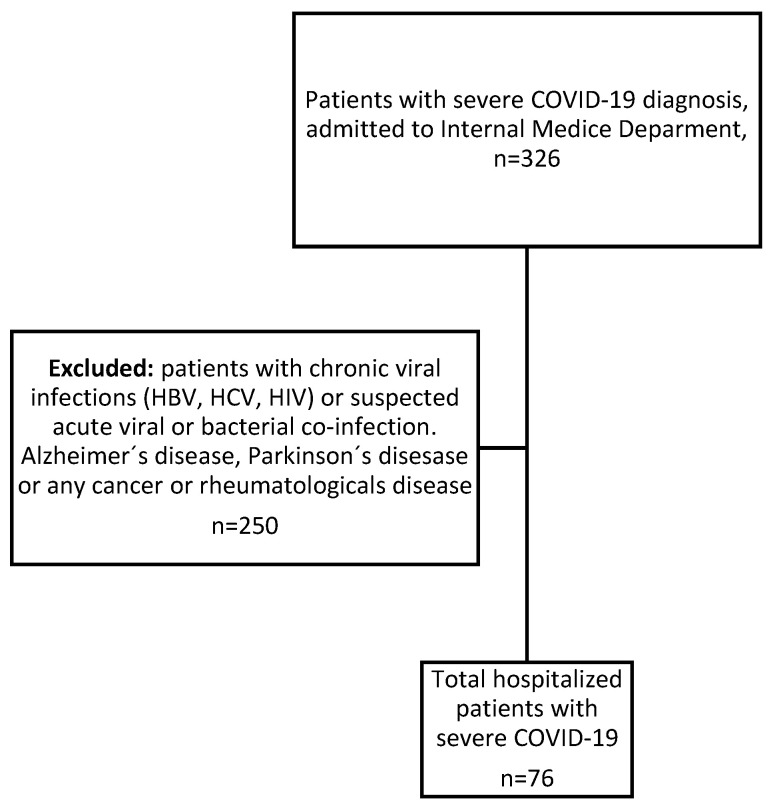
Flowchart of patient selection. HBV: Hepatitis B virus; HCV: hepatitis C virus; HIV: Human immunodeficiency virus.

Patients with suspected coinfection at the time of enrollment were excluded based on predefined clinical and laboratory criteria. These included radiographic patterns suggestive of bacterial pneumonia, leukocytosis with neutrophilia inconsistent with viral inflammation, elevated procalcitonin levels, or positive bacterial cultures obtained within the first 48 h of hospitalization. Microbiological records were reviewed to minimize misclassification. Additionally, patients with neurodegenerative diseases, chronic viral infections, rheumatological conditions, or cancer were excluded to reduce potential confounding effects on *OAS* gene expression [39,40,41] (Figure 1).

Healthy subjects (HS) were matched by age and sex to enable a comparison of cytokine levels and gene expression. All healthy subjects were enrolled prior to the COVID-19 pandemic; therefore, SARS-CoV-2 exposure and vaccination status were not applicable.

### 2.3. SARS-CoV-2 Diagnosis

Upon admission, viral RNA was extracted from nasopharyngeal samples using the Quick RNA Viral Kits (Zymo Research, Irvine, CA, USA) to confirm the COVID-19 diagnosis by a second RT-PCR using the COVIFLU multiplex kit (Genes2Life, Mexico City, Mexico). Subsequently, SARS-CoV-2 variant identification was confirmed by whole-genome sequencing. This approach was described in our previous study [42].

Viral RNA was isolated from nasopharyngeal swab samples preserved in viral transport medium using the Quick RNA Viral Kit (Zymo Research, Irvine, CA, USA). Library preparation and sequencing were performed using the COVIDSeq Test (Illumina, San Diego, CA, USA) according to the manufacturer’s protocol, which employs multiplex amplification covering the complete SARS-CoV-2 genome. Sequencing runs were conducted on a MiSeq platform (Illumina, San Diego, CA, USA). Raw sequence data were processed and assembled into full viral genomes using the DRAGEN COVID Lineage Workflow available on the Illumina BaseSpace platform.

### 2.4. Data Collection and Clinical Assessment

Sociodemographic characteristics were obtained from electronic medical records and included age (categorized according to prevalence as <40, 40–60, and >60 years), sex, time from symptom onset, presence of comorbidities, vaccination status (complete: one or two doses according to vaccine platform), and treatment prior to hospitalization.

The COVID-GRAM score was calculated to predict the risk of progression to critical illness. It is based on ten parameters: chest radiographic abnormality, age, hemoptysis, dyspnea, unconsciousness, number of comorbidities, cancer history, neutrophil-to-lymphocyte ratio, lactate dehydrogenase, and direct bilirubin [43]. Cytokine levels were also evaluated as clinical parameters, as described below.

Clinical outcomes were defined as hospital discharge (survivor) or in-hospital mortality (non-survivor), which served as the dependent variable. *OAS* gene expression was considered the independent variable. Sample size was determined based on the prevalence of hospitalized patients during the study period. Symptom onset, comorbidities, vaccination status, and treatment prior to hospitalization were considered potential confounding variables. Healthy subjects (HS) matched by age and sex were included for comparative analyses of cytokine levels and gene expression.

### 2.5. Reverse Transcription

Total RNA was extracted from 5 mL of peripheral blood collected from 76 patients and 10 healthy subjects matched by age and sex. Total leukocytes were isolated using the phenol–chloroform method described by Chomczynski and Sacchi [44]. RNA concentration and purity were evaluated by spectrophotometry using a NanoDrop 2000 system (Thermo Scientific, Waltham, MA, USA), and only samples with A260/A280 ratios between 1.9 and 2.1 were processed for reverse transcription, confirming suitable RNA quality.

For mRNA analysis, 1 µg of total RNA was reverse transcribed using oligo (dT) primers and M-MLV reverse transcriptase according to the manufacturer’s instructions (Promega, Madison, WI, USA).

### 2.6. Quantitative mRNA Expression

Quantitative PCR (qPCR) was performed using *TaqMan*™ Gene Expression Assays (Thermo Fisher Scientific, Applied Biosystems, Waltham, MA, USA). The following assays were used: *OAS1* (Cat: #4331182, ID: Hs00973635_m1), *OAS2* (Cat: #4331182, ID: Hs00942643_m1), *OAS3* (Cat: #4331182, ID: Hs00196324_m1) and *OAS-L* (Cat: #4331182, ID: Hs00610058_m1).

Amplification reactions were carried out on a QuantStudio^TM^ 5 Real-Time PCR system (Thermo Fisher Scientific, Applied Biosystems, Waltham, MA, USA). Relative gene expression was normalized to *GAPDH* as the endogenous reference gene, following approaches used in COVID-19 host gene expression studies [45]. Its stability across groups was confirmed by the Kruskal–Wallis test of Ct values (*p* > 0.05). Relative gene expression was calculated using the comparative threshold cycle (2^−ΔΔCt^) method and expressed as log_2_ fold change.

### 2.7. Cytokine Determinations

Peripheral blood samples were obtained from all study participants, and serum was used to quantify cytokine levels. IFN-γ, IL-1β, IL-6, IL-8, MCP-1, and TNF-α were measured using the Bio-Plex^®^ MAGPIX^TM^ system (Human Cytokine 17-Plex Panel, Cat: #M5000031YV). This system employs fluorescently labeled magnetic microspheres, each with a distinct spectral signature. This design allows multiplex cytokine detection.

Additionally, high-sensitivity enzyme-linked immunosorbent assays (ELISAs) were used for specific cytokine quantification. The following kits were employed: Human IL-6 High-Sensitivity ELISA (Cat: #BMS213HS), Human TNF-α High-Sensitivity ELISA (Cat: #BMS223HS), and Human IFN-α ELISA (Cat: #BMS216) (Thermo Fisher Scientific, Applied Biosystems, Waltham, MA, USA). IFN-β levels were determined using the Human Interferon Beta SimpleStep ELISA^®^ Kit (Cat: #Ab278127, Abcam™, Cambridge, UK). All assays were performed according to the manufacturer’s instructions.

### 2.8. Hematological Indices

Peripheral blood samples were collected from all hospitalized patients in EDTA tubes, and complete blood counts were performed using a BC-5150 analyzer (Mindray, Shenzhen, China). Hematological indices, including the neutrophil-to-lymphocyte ratio (NLR) and platelet-to-lymphocyte ratio (PLR), were calculated by dividing absolute neutrophil or platelet counts by absolute lymphocyte counts at admission.

### 2.9. Statistical Analysis

Statistical analyses were performed using GraphPad Prism v5.0 (GraphPad Software, San Diego, CA, USA), SPSS v25.0 (IBM Corp., Armonk, NY, USA), and R software (version 4.5.2.; R Foundation for Statistical Computing, Vienna, Austria). Categorical variables were expressed as frequencies and percentages and compared using the χ^2^ test. Normality of continuous variables was assessed using the Shapiro–Wilk test. Non-normally distributed variables were expressed as medians with 95% confidence intervals. Comparisons among three or more groups were performed using the Kruskal–Wallis test followed by Dunn’s post hoc correction for multiple comparisons. Spearman’s rank correlation coefficient was calculated to assess correlations. Outliers were identified using Grubbs’ test. A *p*-value of <0.05 was considered statistically significant.

### 2.10. Ethical Considerations

This study was approved by the Ethics, Biosafety, and Research Committee of the Centro Universitario de Ciencias de la Salud at the Universidad de Guadalajara (CI-03721) and by the Research Ethics Committee of the Hospital Civil de Guadalajara “Dr. Juan I. Menchaca” (00095). The study was conducted in accordance with the Declaration of Helsinki (2013). All participants provided written informed consent prior to enrollment.

## 3. Results

### 3.1. Clinical and Demographic Characteristics of Hospitalized Patients with Severe COVID-19

A total of 326 hospitalized patients with suspected SARS-CoV-2 infection were initially screened. After qPCR confirmation and application of exclusion criteria, 76 patients were confirmed as severe SARS-CoV-2-positive. These patients were classified as having severe COVID-19. The mean age of the study population was 59 years, with a higher proportion of patients older than 60 years (46.1%).

The mean time from symptom onset to hospital admission was 9 days. No significant correlations were observed between time from symptom onset and *OAS* gene expression levels or circulating cytokine concentrations (Supplementary Table S1). More than 65% of patients presented at least one comorbidity. These included systemic arterial hypertension, diabetes mellitus, chronic kidney disease, chronic heart failure, cerebrovascular disease, chronic obstructive pulmonary disease (COPD), asthma, and smoking. Additionally, 63.2% of patients were not vaccinated against SARS-CoV-2 at the time of hospitalization. Overall, in-hospital mortality was 32.9%, with male patients accounting for 60% of deaths. Notably, among patients who died during hospitalization, 48% were infected with the Omicron variant. Prior to hospital admission, 51.3% of patients had received corticosteroid treatment.

The clinical and sociodemographic characteristics of hospitalized patients with severe COVID-19 are summarized in Table 1.

### 3.2. OAS Gene Expression According to SARS-CoV-2 Variants (Delta and Omicron)

**Figure 2:**
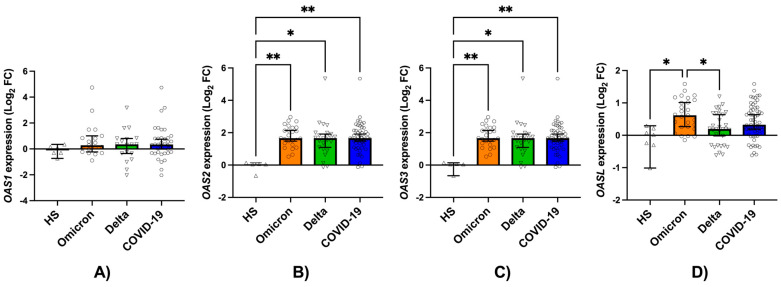
Relative expression levels of *OAS* genes in hospitalized patients with severe COVID-19 according to SARS-CoV-2 viral variant. Relative mRNA expression levels of (**A**) *OAS1*, (**B**) *OAS2*, (**C**) *OAS3*, and (**D**) *OAS-L* are shown in patients infected with the Delta variant, the Omicron variant, and in the combined COVID-19 group. Data are expressed as log_2_ fold change relative to healthy subjects (HS). Bars represent the median and 95% CI. Statistical analysis was performed using the Kruskal–Wallis test followed by Dunn’s post hoc correction for multiple comparisons. Statistical significance is indicated as: * *p* < 0.0001; ** *p* < 0.0002.

Comparison of the overall cohort of hospitalized patients with severe COVID-19 revealed significant induction of *OAS1*, *OAS2*, and *OAS3* gene expression compared with healthy subjects (HS). When expressed as log_2_ fold change (median [95% IC]), *OAS2* showed 4.312 [4.161–4.602] (*p* = 0.0040 vs. HS) and *OAS3* showed 1.660 [1.485–1.916] (*p* = 0.0040 vs. HS) (Figure 2B,C). No significant differences were observed for *OAS1* (0.3444 [-0.01258 to 0.7490]; *p* > 0.9999 vs. HS) (Figure 2A).

When patients infected with the Delta and Omicron variants were compared directly, no statistically significant differences were observed in the expression levels of *OAS1*, *OAS2*, or *OAS3*. For *OAS2*, the log_2_ fold change was 4.554 [4.251–4.743] in Delta versus 4.312 [4.161–4.602] in Omicron (*p* > 0.9999). For *OAS3*, values were 1.659 [1.093–1.916] versus 1.660 [1.480–2.141] (*p* > 0.9999) (Figure 2B,C).

In contrast, *OAS-L* expression was significantly higher in patients infected with the Omicron variant than in those infected with Delta (log_2_ fold change: 0.6169 [0.2653–1.015] vs. 0.1944 [−0.01830 to 0.6355]; *p* = 0.0286) (Figure 2D).

### 3.3. OAS Gene Expression and Clinical Outcome

**Figure 3:**
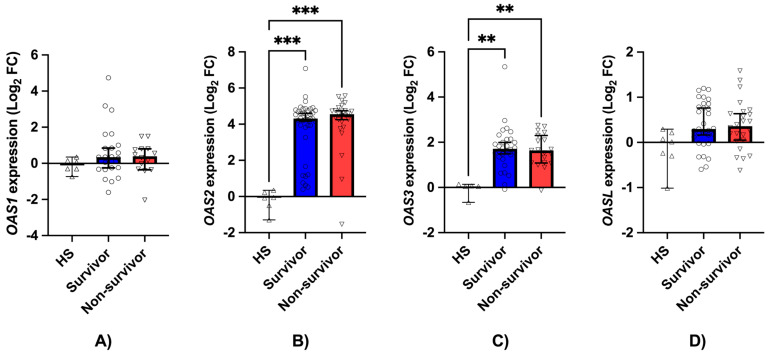
Relative expression levels of *OAS* genes in hospitalized patients with severe COVID-19. (**A**) *OAS1*, (**B**) *OAS2*, (**C**) *OAS3* and (**D**) *OAS-L* are shown as log_2_ fold change relative to healthy subjects (HS). HS: healthy subjects; Survivors: medically discharged patients; Non-survivors: in-hospital mortality. Data are presented as individual data points overlaid on boxplots (median with 95% CI). Statistical analysis was performed using the Kruskal–Wallis test followed by Dunn’s post hoc correction for multiple comparisons. Statistical significance is indicated as: ** *p* < 0.0002; *** *p* < 0.0001.

*OAS1* expression levels were similar between survivors and non-survivors. The log_2_ fold change for *OAS1* was 0.3444 [−0.2501 to 0.8420] in survivors and 0.3931 [−0.3514 to 0.8020] in non-survivors, with no significant differences compared with HS (*p* = 0.5462 and *p* = 0.7942, respectively) (Figure 3A).

In contrast, *OAS2* and *OAS3* expression levels were significantly increased in both survivors and non-survivors compared with HS. For *OAS2*, the log_2_ fold change was 4.312 [4.161–4.602] in survivors (*p* = 0.0009 vs. HS) and 4.554 [4.251–4.743] in non-survivors (*p* = 0.0002 vs. HS) (Figure 3B). For *OAS3*, the log_2_ fold change was 1.711 [1.485–1.990] in survivors (*p* = 0.0025 vs. HS) and 1.640 [1.081–2.301] in non-survivors (*p* = 0.0061 vs. HS) (Figure 3C). No significant differences were observed between survivors and non-survivors for both genes (*p* > 0.9999 for both).

No significant differences in *OAS-L* expression were observed among the groups. Values were 0.2948 [0.1635–0.7605] in survivors and 0.3599 [0.05063–0.6355] in non-survivors. Comparisons with HS were not significant (*p* = 0.0998 for HS vs. survivors, and *p* = 0.1486 for HS vs. non-survivors), nor was the comparison between survivors and non-survivors (*p* > 0.9999) (Figure 3D).

No statistically significant differences in OAS gene expression were observed between vaccinated and unvaccinated patients (all *p* > 0.05; Supplementary Figure S1).

### 3.4. Clinical Outcome and Association with Cytokine Levels

**Figure 4:**
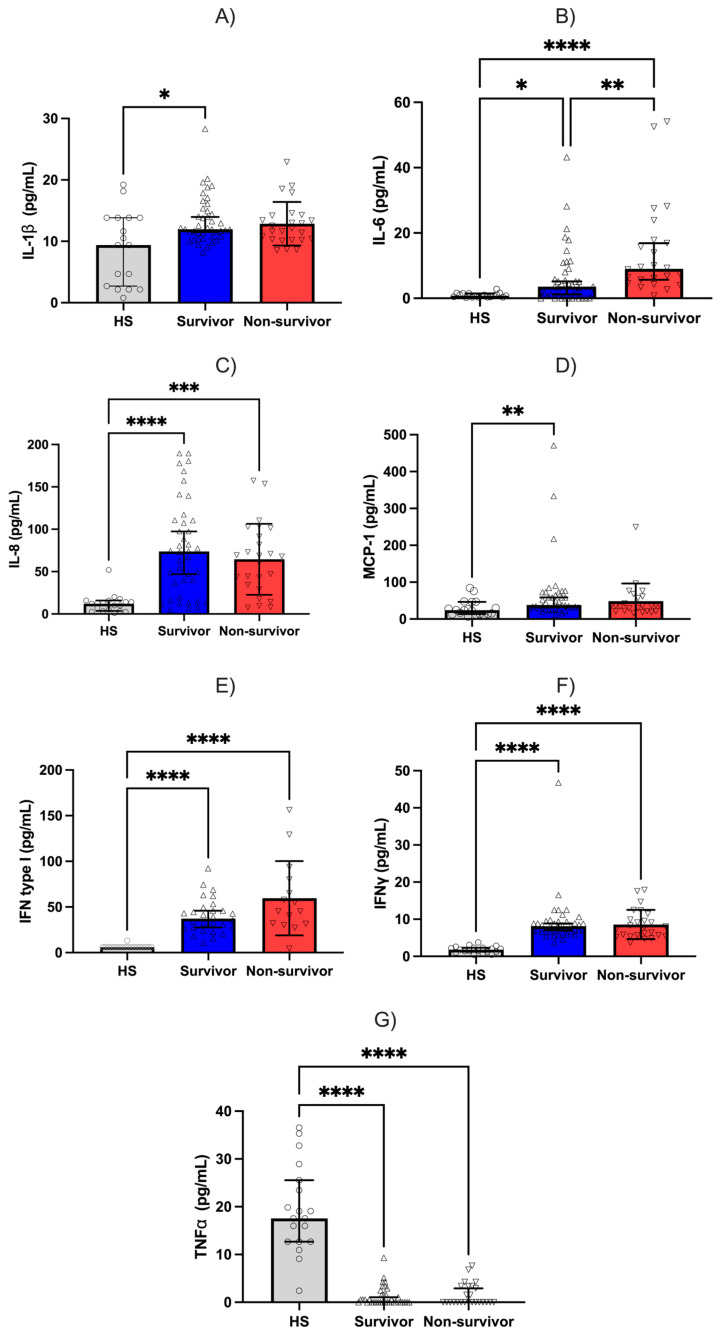
Cytokine levels according to clinical outcome in hospitalized patients with severe COVID-19 and healthy subjects. (**A**) IL-1β, (**B**) IL-6, (**C**) IL-8, (**D**) MCP-1, (**E**) type I interferon (IFN-α/β), (**F**) IFN-γ, and (**G**) TNF-α. Data are expressed as median with 95% IC. Statistical comparisons were performed using the Kruskal–Wallis followed by Dunn’s post hoc correction for multiple comparisons. Statistical significance is indicated as: * *p* < 0.05, ** *p* < 0.01, *** *p* < 0.001, and **** *p* < 0.0001.

Serum levels of IL-1β, IL-6, IL-8, MCP-1, IFN-I (IFN-α and IFN-β), IFN-γ, and TNF-α were compared between survivors and non-survivors. Median IL-1β concentrations were similar between survivors and non-survivors (11.96 pg/mL vs. 12.10 pg/mL, respectively; *p* = 0.5) (Figure 4A). Likewise, IL-8 levels did not differ significantly between groups (median values: 74.32 pg/mL in survivors vs. 67.96 pg/mL in non-survivors; *p* = 0.36) (Figure 4C).

In contrast, serum levels of IL-6, IFN-γ, and IL-8 were significantly elevated in patients who died compared with healthy subjects. Median IL-6 concentrations were 3.56 pg/mL in survivors and 9.64 pg/mL in non-survivors (*p* < 0.0001). IFN-γ levels were 8.12 pg/mL in survivors and 7.79 pg/mL in non-survivors (*p* < 0.0001). IL-8 concentrations were also significantly higher compared with HS. Median values were 74.32 pg/mL in survivors and 67.96 pg/mL in non-survivors (*p* < 0.0002). IL-1β levels were significantly higher in survivors than in healthy subjects (median: 11.96 vs. 9.38 pg/mL; *p* = 0.049). No differences were observed between other group comparisons (Figure 4A–C,G).

**Figure 5:**
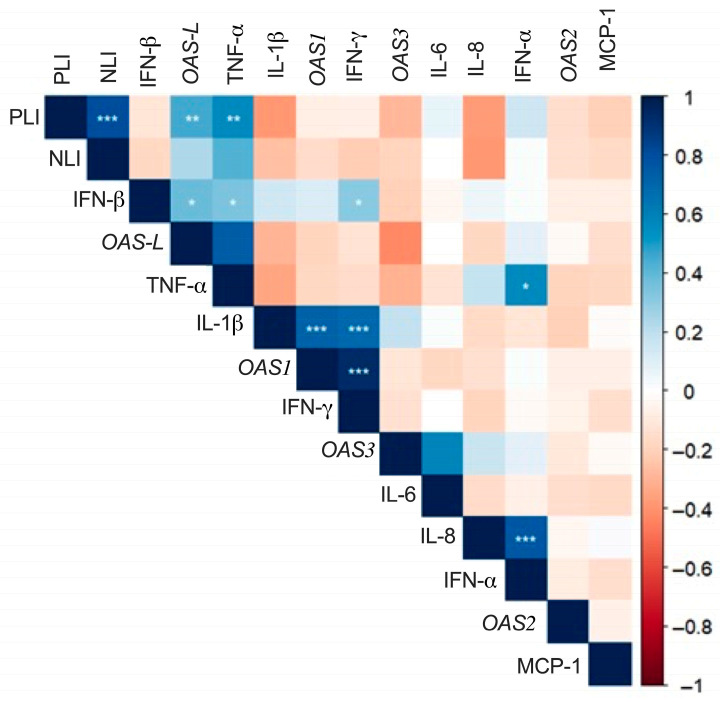
Correlation matrix of *OAS* gene expression, cytokine levels, and hematological indices in hospitalized patients with severe COVID-19. The heatmap illustrates pairwise correlations between *OAS* gene expression (*OAS1*, *OAS2*, *OAS3*, and *OAS-L*), circulating cytokines (IL-1β, IL-6, IL-8, TNF-α, IFN-α, IFN-β, IFN-γ, and MCP-1), and hematological indices, including the neutrophil-to-lymphocyte index (NLI) and platelet-to-lymphocyte index (PLI). Color intensity indicates the strength and direction of correlations (blue: positive; red: negative). Correlations were assessed using Spearman’s rank correlation test. Statistical significance is denoted by asterisks (* *p* < 0.05; ** *p* < 0.01; *** *p* < 0.001).

A correlation analysis was performed among *OAS* gene expression, cytokine levels, and hematological indices (Figure 5). A strong positive correlation was observed between IL-1β and *OAS1* (*p* < 0.001). Moderate positive correlations were identified between IFN-β and *OAS1* (*p* < 0.05). A similar correlation was observed between IFN-β and *OAS-L* (*p* < 0.01). In contrast, most remaining cytokines exhibited weak or non-significant correlations with *OAS* gene expression, suggesting a functional behavior distinct from that of the canonical interferon response.

Multiple linear regression models adjusted for age, sex, comorbidities, and vaccination status were performed to identify factors associated with *OAS* gene expression (ΔΔCt) (Table 2). No significant associations were observed between any host variables and *OAS1*, *OAS2*, *OAS3*, or *OAS-L* expression levels (*p* > 0.05 for all predictors).

## 4. Discussion

In the present study, the expression of *OAS* genes (*OAS1*, *OAS2*, *OAS3*, and *OAS-L*) and circulating cytokine levels were analyzed in 76 Mexican patients hospitalized with severe COVID-19. These patients were infected with the Delta and Omicron variants of SARS-CoV-2. The demographic characteristics of this cohort reinforce the central role of host vulnerability in determining disease severity among hospitalized patients, irrespective of viral lineage.

A national cohort comprising 67,238 hospitalized Mexican patients reported a mean age at hospitalization of 55.29 ± 15.97 years, indicating that hospitalization predominantly occurred in individuals older than 50 years. This age distribution is consistent with that observed in our study and supports existing evidence of an association between increasing age and the risk of hospitalization among Mexican patients with COVID-19 [46]. In the referenced study, advanced age was identified as a variable associated with progression to invasive mechanical ventilation, which in turn was a significant predictor of mortality in hospitalized patients.

Considering advanced age as a major risk factor for COVID-19 hospitalization, aging has been associated with a reduction in circulating plasmacytoid dendritic cells, as well as impaired Toll-like receptor (TLR) function involved in pathogen recognition. These age-related immune alterations are linked to increased susceptibility to infections, including diminished secretion of type I and type III interferons (IFNs). This impairment may facilitate viral acquisition and disease progression [47].

Among the cases analyzed, most patients were infected with the Delta variant. This distribution reflects the epidemiological context in Mexico during late 2021 and early 2022, when Delta predominated alongside the emergence and transitional coexistence of the Omicron variant [37].

The observation that both variants were associated with severe disease in the present study is consistent with international reports indicating that the intrinsically lower virulence of the Omicron variant may be attenuated or even lost in patients of advanced age, with comorbidities, or with incomplete vaccination status—clinical characteristics that were prevalent in our cohort. Consequently, even variants with reduced pulmonary tropism, such as Omicron, can precipitate severe clinical manifestations. Moreover, age behaves as a key determinant of increased risk according to the COVID-GRAM score among patients infected with Omicron, aligning with global evidence showing that the risk of hospitalization associated with Omicron increases substantially in older adults [13]. This finding reinforces the concept that intrinsic host vulnerability may drive progression to critical illness, despite infection with a variant exhibiting lower pulmonary tropism. However, this does not necessarily translate into increased mortality [48].

Regarding cytokine levels, we observed significant differences in IL-6 concentrations, which were higher in non-survivors than in patients who were discharged from the hospital. In a longitudinal cohort study of 203 patients with COVID-19, 78.8% exhibited elevated IL-6 levels (>7 pg/mL), which demonstrated strong predictive capacity for mortality [49,50]. IL-6 emerged as the only cytokine in our cohort that showed statistically significant differences with respect to clinical outcomes; however, its isolated use as a sole biomarker of disease severity remains insufficient.

Elevated levels of cytokines such as IL-6, IL-8, and IFN-γ showed significant differences when comparing hospitalized patients with healthy subjects. Their increase has been consistently associated with the need for mechanical ventilation and mortality. These findings support the concept that severe COVID-19 is not characterized by a classical cytokine storm, but rather by an inflammatory profile predominantly marked by IL-6 dysregulation and alterations in type I interferon responses [6,51,52].

In contrast to the cytokines mentioned above, TNF-α levels were decreased in our patients with HS. This finding may be explained primarily by the use of corticosteroid treatment prior to hospitalization, which can inhibit NF-kB signaling. Additionally, given the mean interval of approximately 9 days between symptom onset and patient recruitment, TNF-α levels may have declined by the time of sampling. Furthermore, higher TNF-α levels have been reported in affected tissues during infections compared with circulating serum levels, which may also contribute to this observation [53].

Despite interferons constituting the first line of antiviral defense, elevated levels of type I IFNs were not observed in patients with severe COVID-19 compared with HS. In contrast, IFN-γ levels were increased in both survivors and non-survivors. Notably, *OAS* gene expression was clearly induced despite the absence of elevated IFN-I levels. This apparent paradox represents a hallmark of severe COVID-19 and may reflect activation of alternative interferon signaling pathways. Type I, II, and III interferons can activate the STAT1/STAT2/IRF9 signaling axis. This activation leads to the formation of the ISGF3 transcriptional complex and induction of interferon-stimulated genes (ISGs). Therefore, *OAS* gene expression in severe COVID-19 may be driven, at least in part, by IFN-γ-mediated signaling rather than canonical IFN-I responses. Although the OAS–RNase L pathway is a well-established component of the interferon-induced antiviral response, during SARS-CoV-2 infection, it is triggered by viral double-stranded RNA replication intermediates, resulting in RNase L activation, viral RNA degradation, and amplification of innate immune signaling pathways that may influence disease progression [54].

In addition, SARS-CoV-2 possesses mechanisms that antagonize the induction and signaling of type I interferons. For example, NSP1 promotes host mRNA degradation and inhibits IFN-I translation, while ORF6 blocks the nuclear translocation of STAT1 and STAT2, impairing interferon signaling. By limiting interferon activation, these viral mechanisms contribute to a blunted IFN-I response in severe disease [55].

A key finding of this study is the significant increase in *OAS2* and *OAS3* expression levels compared with HS. This upregulation may be explained by infection-driven expression in response to SARS-CoV-2. This virus possesses an approximately 29.9 kb RNA genome and generates long double-stranded RNA (dsRNA) intermediates during replication. These long dsRNA structures can be efficiently sensed by *OAS2* and *OAS3*. Notably, *OAS3* exhibits an enhanced functional capacity for detecting dsRNA molecules longer than 50 base pairs, which may account for its pronounced induction during SARS-CoV-2 infection [56,57]. Consistently, experimental murine coronavirus models have shown that infection of myeloid cells is associated with high levels of *OAS* expression, independent of interferon secretion [58]. In contrast, the lack of significant changes in *OAS-L* expression may be explained by its lack of synthase activity [59,60].

The main strengths of this study include the observation of *OAS* gene overexpression despite reduced circulating interferon levels, the exclusive focus on patients hospitalized with severe COVID-19, and the demonstration that this expression pattern was present in infections caused by both Delta and Omicron variants. Additionally, the results were not significantly influenced by demographic characteristics such as age, sex, or comorbidities, supporting the robustness of the observed associations. This variant-independent pattern suggests that *OAS* activation may be driven predominantly by disease severity and host immune responses. It appears to be less influenced by viral genotype.

Nevertheless, several limitations should be acknowledged. These include the use of corticosteroid treatment prior to hospitalization in a subset of patients and the relatively long interval between symptom onset and hospital admission. Although no significant correlations were found with sampling time, single-time-point measurements may not fully capture the kinetics of immune responses. Moreover, the limited sample size, the absence of a comparison group with mild or moderate COVID-19, and incomplete data on prior SARS-CoV-2 infections may restrict the generalizability of the findings. The lack of biological samples to assess intracellular *OAS* enzyme levels represents an additional limitation in fully integrating gene expression with functional protein activity. Finally, evaluation of the *OAS* gene expression in nasal epithelial cells would have provided valuable insights into early antiviral responses at the primary site of viral entry and represents an important direction for future studies.

## 5. Conclusions

In survivor patients, gene expression analysis demonstrated that *OAS2* and *OAS3* were significantly overexpressed compared with uninfected healthy subjects. In contrast, non-survivors exhibited a trend toward lower expression of these genes relative to survivors. This finding suggests a more efficient activation of the endogenous antiviral response mediated by the OAS–RNase L pathway in patients with better clinical outcomes. Importantly, variant-dependent reductions in disease severity reported in community-based studies may not translate to hospitalized populations with high intrinsic risk. Additionally, IL-6 and IL-8 displayed a predominantly proinflammatory profile, further supporting their role as biomarkers of poor prognosis in severe COVID-19.

*OAS* gene expression was markedly increased in severe COVID-19 compared with uninfected individuals, particularly for *OAS2* and *OAS3*. This upregulation was observed in patients infected with both Delta and Omicron variants. In the context of critical illness, activation of this antiviral pathway is driven primarily by the severity of the host response rather than by the specific viral lineage.

Circulating type I interferons (IFN-α and IFN-β) were reduced, whereas IFN-γ levels were elevated and *OAS* gene expression remained high. This pattern suggests early or localized interferon-mediated activation sufficient to induce antiviral genes but not sustained over time or detectable in peripheral circulation.

From a translational perspective, the OAS-RNase L pathway may represent a potential axis for disease prognosis. It may also serve as a target for future therapeutic modulation aimed at enhancing early antiviral immunity while limiting excessive inflammation.

## Figures and Tables

**Table 1:** Clinical and sociodemographic characteristics of hospitalized patients with severe COVID-19.

Variable	Hospitalized Patients (n = 76)
Age (years), mean ± SD	59.28 ± 15.27
Age groups, n (%)	
<40 years	10 (13.2)
40–60 years	31 (40.8)
>60 years	35 (46.1)
Sex, n (%)	
Female	28 (36.8)
Male	48 (63.2)
BMI (kg/m^2^), median (min–max)	31 (21–57)
Days to first symptom, mean ± SD	9.49 ± 3.58
Comorbidities, n (%)	
No	24 (31.6)
Yes	52 (68.4)
Vaccinated, n (%)	
No	48 (63.2)
Yes	28 (36.8)
Vaccination status, n (%)	
Incomplete	5 (17.9)
Complete	23 (82.1)
Corticosteroid use prior to hospitalization, n (%)	
No	37 (48.7)
Yes	39 (51.3)
Clinical outcome, n (%)	
Survivors (hospital discharged)	51 (67.1)
Non-survivors (in-hospital mortality)	25 (32.9)
SARS-CoV-2 variants, n (%)	
Delta	45 (59)
Omicron	31 (41)
COVID-GRAM score, mean ± SD	140 ± 31

SD: standard deviation; Min: minimum; Max: maximum; COVID-GRAM score range: 6–128 (moderate risk).

**Table 2:** Multiple linear regression models evaluating factors associated with *OAS* gene expression.

Adjusted Model	*OAS1* (β, SE)	*OAS2* (β, SE)	*OAS3* (β, SE)	*OAS-L* (β, SE)
Intercept	1.842 (0.611) **	0.933 (0.402) *	1.274 (0.518) *	0.691 (0.356)
Age	−0.321 (0.48)	−0.085 (35.22)	0.126 (0.01)	0.043 (3)
Sex (female)	0.241 (17.49)	0.123 (1093.3)	0.074 (0.59)	0.031 (93.71)
Comorbidities (Yes)	0.129 (17.67)	−0.237 (1129.84)	0.051 (0.6)	−0.124 (98.77)
Vaccinated (Yes)	−0.156 (17.69)	0.247 (1167.08)	0.155 (0.65)	0.045 (103.83)

The dependent variable was *OAS* gene expression expressed as ΔΔCt; β: regression coefficient; SE: standard error. * *p* < 0.05; ** *p* < 0.01.

## Data Availability

The data presented in this study are available on request from the corresponding author.

## Supplementary Materials

The following supporting information can be downloaded at: https://www.mdpi.com/article/10.3390/jcm15062189/s1, Table S1: Correlation between time from symptom onset, OAS gene expression, and cytokine levels; Figure S1: Comparative expression levels of *OAS* family genes in hospitalized patients with severe COVID-19 according to SARS-CoV-2 variant. Bars represent relative mRNA expression of *OAS1*, *OAS2*, *OAS3*, and *OAS-L* in patients infected with the Delta and Omicron variants. Data are shown as median values. Differences between variants were analyzed using non-parametric tests, and statistical significance was established at *p* < 0.05.

## Abbreviations

The following abbreviations are used in this manuscript:

ARDS	Acute respiratory distress syndrome
BMI	Body mass index
dsRNA	Double-stranded RNA
ELISA	Enzyme-linked immunosorbent assay
HS	Healthy subjects
IFN	Interferon
IL	Interleukin
IMV	Invasive mechanical ventilation
ISGs	Interferon-stimulated genes
Med	Median
NLR	Neutrophil-to-lymphocyte ratio
OAS	Oligoadenylate synthetase
OAS-L	Oligoadenylate synthetase-like
PLR	Platelet-to-lymphocyte ratio
qPCR	Quantitative polymerase chain reaction
RNase L	Ribonuclease L
SaO_2_	Arterial oxygen saturation
SARS-CoV-2	Severe acute respiratory syndrome coronavirus 2
SD	Standard deviation
VOCs	Variants of concern
WHO	World Health Organization
