# Supplementary material for: Overexpression of OAS Genes in Severe COVID-19: A Cross-Sectional Study of Hospitalized Patients Infected with Delta and Omicron Variants
Source: J Clin Med. 2026 Mar 13;15(6):2189. doi: 10.3390/jcm15062189 (PMC13027319; doi:10.3390/jcm15062189)
Supplement: Supplementary file 1 [file jcm-15-02189-s001.zip › jcm-4151128-supplementary.pdf]

Journal of
Clinical Medicine

MDPI

Supplementary information

Table S1. Correlation between time from symptom onset, *OAS* gene expression, and cytokine levels

Variable	Spearman's ϱ	p-value
**OAS genes ($^{\Delta\Delta Ct}$)**		
OAS1	0.068	0.691
OAS2	0.050	0.692
OAS3	0.002	0.986
OAS-L	-0.004	0.978
**Cytokines (pg/mL)**		
IL-1β	-0.066	0.597
IL-6	-0.093	0.492
IL-8	0.087	0.488
MCP-1	0.124	0.329
TNF-α	-0.160	0.221
IFN-α	-0.101	0.513
IFN-β	-0.174	0.204
IFN-γ	0.045	0.722

Time from symptom onset is expressed in days. OAS gene expression was measured by the ΔΔCt method relative to GAPDH

Figure S1. Comparative expression levels of *OAS* family genes in hospitalized patients with severe COVID-19 according to SARS-CoV-2 variant. Bars represent relative mRNA expression of *OAS1*, *OAS2*, *OAS3*, and *OAS-L* in patients infected with the Delta and Omicron variants. Data are shown

 https://doi.org/10.3390/xxxxx

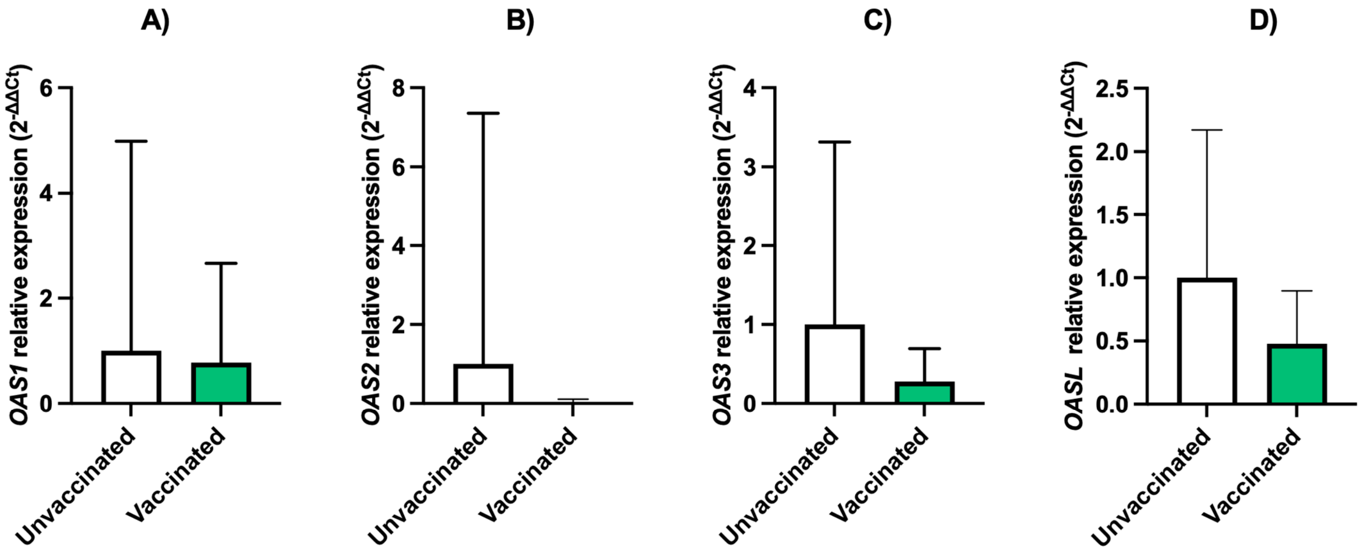

as median values. Differences between variants were analyzed using non-parametric tests, and statistical significance was established at $p < 0.05$.
